# Taxonomic Resolution of the Nematophagous Fungal Isolate ARF18 via Genome Sequencing

**DOI:** 10.1128/genomeA.00903-17

**Published:** 2017-08-24

**Authors:** Sandeep Sharma, Alex Z. Zaccaron, John B. Ridenour, Amy Bradshaw, Terry L. Kirkpatrick, Kelly Cartwright, Burton H. Bluhm

**Affiliations:** a Department of Plant Pathology, University of Arkansas, Division of Agriculture, Fayetteville, Arkansas, USA; b Southern Arkansas University, Magnolia, Arkansas, USA; c Agricultural Research Initiatives, Inc., Fayetteville, Arkansas, USA

## Abstract

The taxonomically uncharacterized nematophagous fungus ARF18, which parasitizes cysts, juveniles, and adults of the soybean cyst nematode (*Heterodera glycines*), was proposed as a nematode biological control agent in 1991. A 46.3-Mb draft genome sequence of this fungus is presented, and a tentative taxonomic identification as a novel species of *Brachyphoris* is proposed.

## GENOME ANNOUNCEMENT

Plant-parasitic nematodes are destructive pathogens of crop plants worldwide and cause estimated losses in excess of $150 billion annually (1). Control of plant-parasitic nematodes relies on chemical nematicides and cultural practices, including crop rotation and using resistant cultivars. The manufacture and use of several key chemical nematicides has been discontinued due to human health risks and environmental concerns. Resistant cultivars do not currently exist for all crops, and effective crop rotation schemes are lacking for many cropping systems due to economic concerns. Alternative nematode control tactics are urgently needed for many major economic crops (2, 3). The hyphomycete fungus ARF18 was first isolated from infected cysts of *Heterodera glycines* nearly 30 years ago (4). Because the fungus parasitizes all stages of the nematode, including eggs, juveniles, and adults in both soil and culture media (5), it was suggested as a potential biological control organism. Culture conditions have not yet been identified that induce conidiation or other morphological features that are required for classical taxonomic identification. Additionally, nothing is yet known about nematophagy in ARF18 at the molecular level.

The genome of ARF18 was sequenced with Pacific Biosciences (PacBio) technology, which generated 142,598 reads. Lengths varied from 35 bp to 43,743 bp with an average length of 7,686 bp. A draft genome assembly of the fungus was obtained with Canu version 1.1 (6), following the program instructions for low coverage data sets. The ARF18 draft genome assembly was improved by merging contigs into scaffolds with AHA from the SMRTanalysis suite version 2.3.0 (http://www.pacb.com/products-and-services/analytical-software/devnet/devnet-analysis-tools). The resulting genome assembly had 46,639,970 bp organized into 412 scaffolds with an *N*_50_ value of 177 kb, an *L*_50_ value of 76, and a GC content of 44.6%. Compared to the genome of *Arthrobotrys oligospora* and many other ascomycetes, ARF18 had a slightly larger genome (7, 8).

Gene prediction was performed with the Maker version 2.31.6 pipeline (9) with homology evidence proteins from *A. oligospora* ATCC 24927, *Monacrosporium haptotylum* CBS 200.50, and *Drechslerella stenobrocha* 248. A total of 14,461 protein-encoding genes, with an average length of 1,028 bp, were predicted in the ARF18 genome assembly. Through BLAST analyses, several genes were identified that could play roles in nematode pathogenesis, including cuticle-degrading serine proteases, alkaline serine proteases, and chitinases (10–12). Further examination of the ITS1-5.8s to ITS2 rDNA region suggested that ARF18 belongs to a distinct monophylogenetic clade within *Brachyphoris*, a genus of nematophagous fungi that belongs to the ascomycete family *Orbiliaceae* (13–15). Based on BLAST analyses, most of the genes analyzed showed high identity to *A. oligospora* and *Dactylellina haptotyla*, both nematophagous fungi within the *Orbiliaceae* family, supporting the taxonomic placement of ARF18 within the *Orbiliaceae* family.

Currently, only a few nematophagous fungal genomes are publicly available. Thus, the genome sequence of this fungus will provide a useful resource to study the biology of nematophagous fungi, especially within the *Brachyphoris* genus. Further analyses of the genome of ARF18 will also provide important information regarding the molecular basis of fungal nematophagy and guide the potential development of this nematode pathogen as a biological control agent.

### Accession number(s).

This whole-genome shotgun project has been deposited at DDBJ/ENA/GenBank under the accession no. AZLU00000000. The version described in this paper is the first version, AZLU01000000.

## References

[1] Abad P, Gouzy J, Aury JM, Castagnone-Sereno P, Danchin EGJ, Deleury E, Perfus-Barbeoch L, Anthouard V, Artiguenave F, Blok VC, Caillaud MC, Coutinho PM, Dasilva C, De Luca F, Deau F, Esquibet M, Flutre T, Goldstone JV, Hamamouch N, Hewezi T, Jaillon O, Jubin C, Leonetti P, Magliano M, Maier TR, Markov GV, McVeigh P, Pesole G, Poulain J, Robinson-Rechavi M, Sallet E, Ségurens B, Steinbach D, Tytgat T, Ugarte E, van Ghelder C, Veronico P, Baum TJ, Blaxter M, Bleve-Zacheo T, Davis EL, Ewbank JJ, Favery B, Grenier E, Henrissat B, Jones JT, Laudet V, Maule AG, Quesneville H, Rosso M-N, Schiex T, Smant G, Weissenbach J, Wincker P 2008 Genome sequence of the metazoan plant-parasitic nematode meloidogyne incognita. Nat Biotechnol 26:909–915. doi:10.1038/nbt.1482. 18660804

[2] Oka Y, Koltai H, Bar-Eyal M, Mor M, Sharon E, Chet I, Spiegel Y 2000 New strategies for the control of plant-parasitic nematodes. Pest Manag Sci 56:983–988. doi:10.1002/1526-4998(200011)56:11<983::AID-PS233>3.0.CO;2-X.

[3] Tian B, Yang J, Zhang KQ 2007 Bacteria used in the biological control of plant-parasitic nematodes: populations, mechanisms of action, and future prospects. FEMS Microbiol Ecol 61:197–213. doi:10.1111/j.1574-6941.2007.00349.x. 17651135

[4] Kim DG, Riggs RD 1991 Characteristics and efficacy of a sterile hyphomycete (ARF18), a new biocontrol agent for *Heterodera glycines* and other nematodes. J Nematol 23:275–282. 19283127 PMC2619172

[5] Timper P, Riggs RD, Crippen DL 1999 Parasitism of sedentary stages of *Heterodera glycines* by isolates of a sterile nematophagous fungus. Phytopathology 89:1193–1199. doi:10.1094/PHYTO.1999.89.12.1193. 18944645

[6] Koren S, Walenz BP, Berlin K, Miller JR, Bergman NH, Phillippy AM 2017 Canu: scalable and accurate long-read assembly via adaptive *k*-mer weighing and repeat separation. Genome Res 27:722–736. doi:10.1101/gr.215087.116. 28298431 PMC5411767

[7] Yang J, Wang L, Ji X, Feng Y, Li X, Zou C, Xu J, Ren Y, Mi Q, Wu J, Liu S, Liu Y, Huang X, Wang H, Niu X, Li J, Liang L, Luo Y, Ji K, Zhou W, Yu Z, Li G, Liu Y, Li L, Qiao M, Feng L, Zhang KQ 2011 Genomic and proteomic analyses of the fungus *Arthrobotrys oligospora* provide insights into nematode-trap formation. PLoS Pathog 7:e1002179. doi:10.1371/journal.ppat.1002179. 21909256 PMC3164635

[8] Galagan JE, Calvo SE, Borkovich KA, Selker EU, Read ND, Jaffe D, FitzHugh W, Ma LJ, Smirnov S, Purcell S, Rehman B, Elkins T, Engels R, Wang SG, Nielsen CB, Butler J, Endrizzi M, Qui DY, Ianakiev P, Bell-Pedersen DB, Nelson MA, Werner-Washburne M, Selitrennikoff CP, Kinsey JA, Braun EL, Zelter A, Schulte U, Kothe GO, Jedd G, Mewes W, Staben C, Marcotte E, Greenberg D, Roy A, Foley K, Naylor J, Stange-Thomann N, Barrett R, Gnerre S, Kamal M, Kamvysselis M, Mauceli E, Bielke C, Rudd S, Frishman D, Krystofova S, Rasmussen C, Metzenberg RL, Perkins DD, Kroken S, Cogoni C, Macino G, Catcheside D, Li WX, Pratt RJ, Osmani SA, DeSouza CPC, Glass L, Orbach MJ, Berglund JA, Voelker R, Yarden O, Plamann M, Seiler S, Dunlap J, Radford A, Aramayo R, Natvig DO, Alex LA, Mannhaupt G, Ebbole DJ, Freitag M, Paulsen I, Sachs MS, Lander ES, Nusbaum C, Birren B 2003 The genome sequence of the filamentous fungus *Neurospora crassa*. Nature 422:859–868. doi:10.1038/nature01554. 12712197

[9] Huang XW, Zhao NH, Zhang KQ 2004 Extracellular enzymes serving as virulence factors in nematophagous fungi involved in infection of the host. Res Microbiol 155:811–816. doi:10.1016/j.resmic.2004.07.003. 15567274

[10] Lopez-Llorca LV, Gómez-Vidal S, Monfort E, Larriba E, Casado-Vela J, Elortza F, Jansson HB, Salinas J, Martín-Nieto J 2010 Expression of serine proteases in egg-parasitic nematophagous fungi during barley root colonization. Fungal Genet Biol 47:342–351. doi:10.1016/j.fgb.2010.01.004. 20097301

[11] Yang J, Tian B, Liang L, Zhang KQ 2007 Extracellular enzymes and the pathogenesis of nematophagous fungi. Appl Microbiol Biotechnol 75:21–31. doi:10.1007/s00253-007-0881-4. 17318531

[12] Yang J, Yu Y, Li J, Zhu W, Geng Z, Jiang D, Wang Y, Zhang KQ 2013 Characterization and functional analyses of the chitinase-encoding genes in the nematode-trapping fungus Arthrobotrys oligospora. Arch Microbiol 195:453–462. doi:10.1007/s00203-013-0894-6. 23661195

[13] Chen J, Xu L-L, Liu B, Liu X-Z 2007 Taxonomy of *Dactylella* complex and *Vermispora*. I. Generic concepts based on morphology and ITS sequences data. Fungal Divers 26:73–83.

[14] Chen J, Xu L-L, Liu B, Liu X-Z 2007 Taxonomy of *Dactylella* complex and *Vermispora*. II. The genus *Dactylella*. Fungal Divers 26:85–126.

[15] Chen J, Xu L-L, Liu B, Liu X-Z 2007 Taxonomy of *Dactylella* complex and *Vermispora*. III. A new genus *Brachyphoris* and revision of *Vermispora*. Fungal Divers 26:127–142.

